# Defining the roles of local precipitation and anthropogenic water sources in driving the abundance of *Aedes aegypti*, an emerging disease vector in urban, arid landscapes

**DOI:** 10.1038/s41598-023-50346-3

**Published:** 2024-01-24

**Authors:** Erica A. Newman, Xiao Feng, Jesse D. Onland, Kathleen R. Walker, Steven Young, Kirk Smith, John Townsend, Dan Damian, Kacey Ernst

**Affiliations:** 1 Department of Ecology and Evolutionary Biology, University of Arizona, Tucson, AZ 85721 USA; 2 Present Address: Department of Integrative Biology, University of Texas at Austin, Austin, TX 78712 USA; 3 Department of Biology, University of North Carolina, Chapel Hill, NC 27599 USA; 4 Independent Analyst, Kitchener, ON Canada; 5 Department of Entomology, University of Arizona, 1140 E South Campus Drive, Forbes 410, Tucson, AZ 85721 USA; 6 Maricopa County Environmental Services Vector Control Division, 3220 W Gibson Ln, Phoenix, AZ 85009 USA; 7 Maricopa County Office of Enterprise Technology, 301 S 4Th Ave #200, Phoenix, AZ 85003 USA; 8 Mel and Enid Zuckerman College of Public Health, University of Arizona, Tucson, AZ 85721 USA

**Keywords:** Environmental sciences, Diseases, Entomology, Ecology, Climate-change ecology, Ecological modelling, Invasive species, Population dynamics, Urban ecology

## Abstract

Understanding drivers of disease vectors’ population dynamics is a pressing challenge. For short-lived organisms like mosquitoes, landscape-scale models must account for their highly local and rapid life cycles. *Aedes aegypti*, a vector of multiple emerging diseases, has become abundant in desert population centers where water from precipitation could be a limiting factor. To explain this apparent paradox, we examined *Ae. aegypti* abundances at > 660 trapping locations per year for 3 years in the urbanized Maricopa County (metropolitan Phoenix), Arizona, USA. We created daily precipitation layers from weather station data using a kriging algorithm, and connected localized daily precipitation to numbers of mosquitoes trapped at each location on subsequent days. Precipitation events occurring in either of two critical developmental periods for mosquitoes were correlated to suppressed subsequent adult female presence and abundance. LASSO models supported these analyses for female presence but not abundance. Precipitation may explain 72% of *Ae. aegypti* presence and 90% of abundance, with anthropogenic water sources supporting mosquitoes during long, precipitation-free periods. The method of using kriging and weather station data may be generally applicable to the study of various ecological processes and patterns, and lead to insights into microclimates associated with a variety of organisms’ life cycles.

## Introduction

### Localized environmental data can be linked to disease vectors’ life cycles

Predicting disease vector dynamics is of considerable importance for human and wildlife health globally, and is becoming increasingly urgent with global climate and land use changes^1,2^. Understanding the drivers of disease vectors’ abundances and range expansions, as well as their life cycles, population structures, and interaction with human-modified environments will have immediate applications for prediction of disease exposure and transmission^3^, public health, and intervention efforts^4–7^. However, one of the major challenges in predicting population-level dynamics of disease vectors is defining and incorporating the appropriate data to represent the relevant spatial and temporal extents of complex processes and ecological interactions^8–10^.

In disease ecology, the mechanisms relating both climate and weather to their effects on organisms at the appropriate scales are often poorly studied, and rely on lab- rather than field-based studies^11^. Relevant environmental conditions are often short-term, variable, and highly localized, and appropriate statistical approaches to connect local-scale weather information to organisms’ life cycles may be lacking^12^ (but see^13,14^). Although laboratory experiments^15,16^ can help define the causal relationships between weather variables and organismal biology, they do not translate directly to landscape-level or regional predictions. Many spatial models of disease vector establishment and spread therefore rely on coarse-resolution climate predictors over broad extents, such as recent attempts employing climate envelope approaches^17,18^. These studies demonstrate that broad distributional patterns may be predictable with high error, and that climate envelope models applied over large extents fail to match the resolution of fine-scale processes and patterns. These may include weather changes, microclimates, as well as environmental variability related to structure, weather, species interactions^11,19–21^, and reproductive success^22^. Climate envelope approaches may be inappropriate for modeling emerging diseases when system-specific knowledge is ignored^3,23^ or the goal is to predict population densities^24^. Fine-scale environmental data is therefore necessary for testing hypotheses linking spatially- and temporally- structured population dynamics to underlying ecological variation^25,26^.

**Figure 1:**
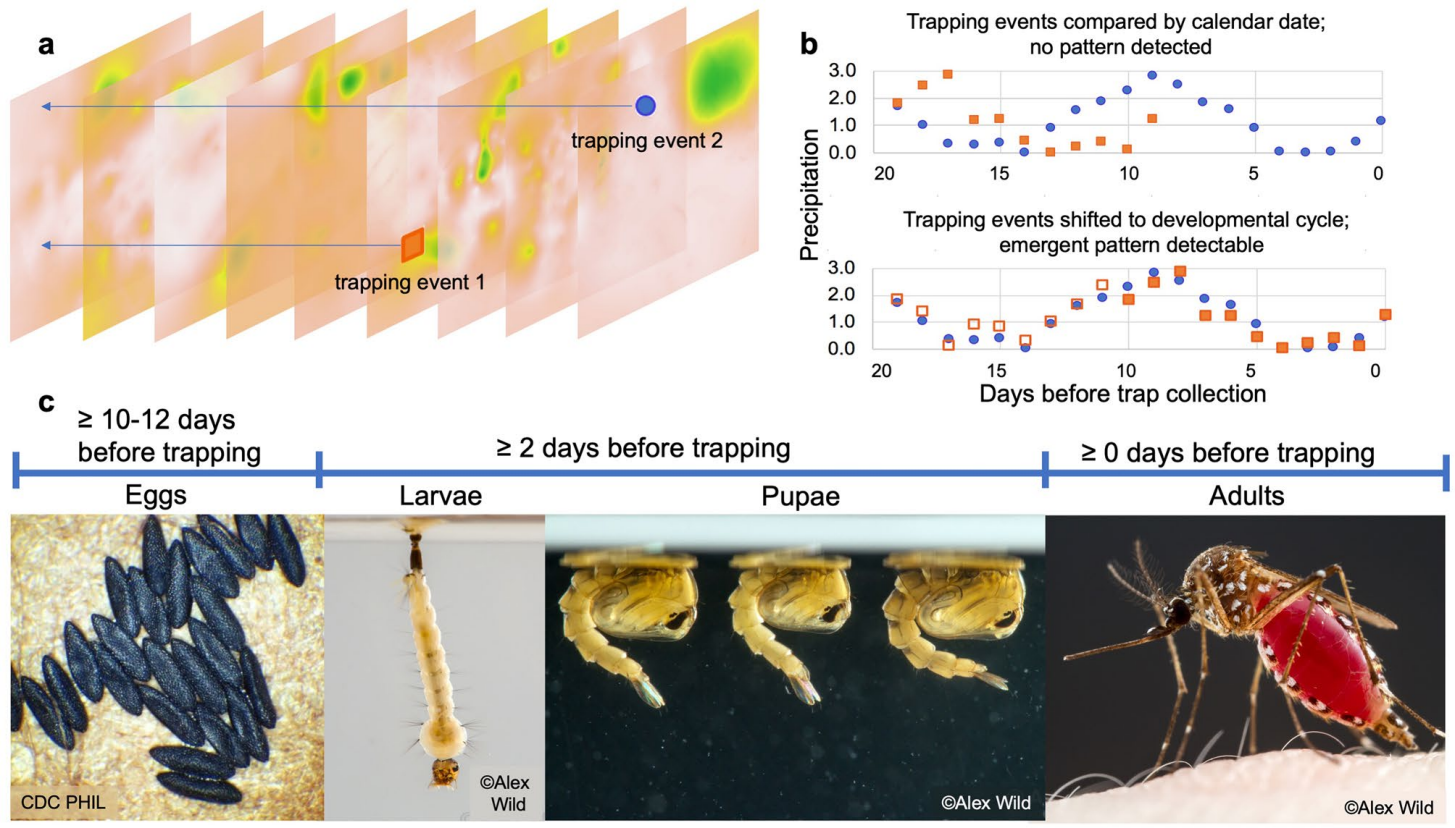
Realigning precipitation data to the trap collection date for *Ae. aegypti* mosquitoes. (**a**) Mosquitoes are trapped on different days (represented by raster layers of interpolated precipitation) at different locations. Here, trapping event 1 at location 1 is shown as an orange square, and trapping event 2 at location 2 is shown as a blue circle. Arrows indicate the series of highly localized precipitation data each day leading up to the trapping event. Although a limited amount of data extraction is illustrated, all trapping events are associated with 20 days of prior precipitation. (**b**) Here, we align trapping event 1 (in orange) with trapping event 2 (in blue) by the mosquito trapping date, rather than calendar date. This daily precipitation reconstruction and realignment was done for all 100,757 trapping events in the study. We interpret the resulting patterns as they are relevant to **(c)**, the well-established developmental stages and life cycle of *Ae. aegypti*. Photographs copyright Alex Wild and Centers for Disease Control and Prevention, used with permission under CC BY.

The urban, peridomestic mosquito *Ae. aegypti* (L.) (Diptera: Culicidae) is the primary vector of several major diseases, including the arboviruses dengue, Zika, chikungunya, and yellow fever. A highly invasive species originating from Africa, *Ae. aegypti* is now established throughout tropical and semitropical regions of the world^27–29^, and is expanding into the United States^22,30,31^. As the species is strongly anthropophilic, its distribution is linked to urban environments and clustered human dwellings in rural areas^32^. Although temperature responses and survival limits have been extensively studied for *Ae. aegypti* (e.g.,^33,34^), there may be other limits on the abundance of mosquitoes that depend on water availability. Less is known about the effects of environmental factors other than temperature on *Ae. aegypti*^16^, such as seasonal and cumulative precipitation, and the importance of individual rainfall events to their choices of oviposition sites, larval development, and subsequent emergence. Precipitation is known to be an important factor at global, regional, and local scales, e.g.,^35–37^. However, it is still unknown if lack of precipitation can limit population density and overall abundance of *Ae. aegypti* mosquitoes, in part because water from anthropogenic sources can provide sufficient resources for container breeding mosquitoes^38^. Understanding the basic role of precipitation in driving mosquito abundance at fine spatial and temporal scales in conjunction with knowledge of the life cycle of the mosquito can reveal where and when anthropogenic water sources become important (Fig. 1).

### Life history of *Aedes aegypti*

The life history and population biology of *Ae. aegypti* is dependent on water availability. Female mosquitoes lay their eggs in small water containers, and do not oviposit in large, permanent water bodies, irrigation ditches or temporary, shallow pools of water^27^. This species has several traits that may result from adaptation to arid environments, for example, *Ae. aegypti* eggs can survive desiccation for months to years, and persistent water is not necessary^39^. Hatching of eggs is triggered by inundation by water. Progression through life stages for *Ae. aegypti* is temperature-dependent, requiring temperatures between 16 °C, and 34 °C for successful development^27,34^, with the transition from hatching to adult emergence occurring in as few as 7 days at higher temperatures^40^. Adults generally do not disperse beyond 30–60 m from their hatch site and tend to cluster around homes, but will rarely disperse as far as 500 m for oviposition sites^41–43^. The close association between *Ae. aegypti* and humans has allowed the species to establish in otherwise inhospitable climates, relying on human-created water sources such as stored drinking water for larval development^44–46^.

In Maricopa County, Arizona, USA (including metropolitan Phoenix), urbanization has led to human uses of water that create favorable conditions for mosquitoes. Anthropogenic uses of water have reduced the aridity of the local metropolitan area compared to the surrounding Sonoran Desert environment^47^. Urban microclimates have altered temperature, humidity, and availability of oviposition sites^48,49^ including in stormwater drains^50–53^, which constitute refugia for mosquito populations in desert cities. While monitoring natural rainfall events is common, there are no comprehensive measurements of the large amounts of surface water generated by activities such as “urban greening”: landscape maintenance, intentional flooding of lawns, and watering of ornamental plants; as well as recreational uses, ornamental features known to be important breeding sites^49^, car washing, and flooding fields for agriculture. Based on biological requirements, we expect that precipitation could be a limiting factor in mosquito activity. If lack of precipitation does not limit mosquito activity at trapping locations, mosquito breeding habitat is only available from the only other source of water: anthropogenic uses.

### Precipitation and *Aedes aegypti* abundance

We address how daily and cumulative precipitation affect mosquito abundance where piped water is available. We build our hypotheses off of a useful functional relationship proposed for regions that store drinking water, which specifies that increasing amounts of rainfall will have a complicated but deterministic, non-monotonic relationship with the abundance of adult *Ae. aegypti* mosquitoes^11,54^. The abundance of adult mosquitoes is expected to decrease with increasing precipitation and less need for stored water^38^; then increase along with additional precipitation and habitat formation; and then decrease with further precipitation flushing developing larvae out of containers^55,56^. This conceptual model provides important baseline expectations consistent with the biology of the container-breeding *Ae. aegypti*, but does not distinguish between precipitation from individual rainfall events, and total accumulated precipitation prior to mosquito emergence. From this conceptual model, we expect that female *Ae. aegypti* presence and abundance will be strongly influenced by cumulative precipitation.

We additionally expect that the timing of individual precipitation events will be important to the subsequent abundance of mosquitoes. Because only females transmit arboviruses, we focus on them. Related to their life cycle, *Ae. aegypti* counts 1-6 days following rainfall events should correspond to increased activity of adult females, whereas abundance from 7 to 15 days following rainfall events may additionally correspond to the emergence of new adults^27,40^ due to the local development of eggs into adult mosquitoes. Alternatively, if we find that mosquito abundance is constant after rainfall, this would imply that oviposition sites are available at all times and are not a limiting factor. *Ae. aegypti* numbers may even decrease 5–20 days following a rainfall event if large amounts of rain, such as from monsoon storms, flush away developing larvae from water-filled containers that had contained immature life stages^57^.

A broad network of mosquito traps employed in this study catches mosquitoes from newly emerged to long-lived adult females. These may live slightly longer than 20 days in the wet tropics^58^, but their lifespan in the Sonoran Desert is expected to be shorter due to drier conditions, and dependent on anthropogenic water for survival as well as breeding. By matching trap locations to spatially interpolated precipitation data at those sites for 20 days, counting backwards from the trap collection date, we were able to test two explicit hypotheses for presence and abundance:

#### H1

Adult mosquito presence and abundance (measured by female counts in traps) is limited by *daily precipitation from days prior to and after the egg-laying period*. To assess this, we associated all trapping events with daily precipitation data at the trapping location on each previous day leading up to the trap collection date.

#### H2

Adult mosquito presence and abundance (females only) is limited by *accumulated precipitation prior to and after the egg-laying period* in the vicinity of the trapping location. Cumulative precipitation was calculated as the sum of the spatially interpolated precipitation at the trapping location for 10 and 20 days. Here, abundance will represent both the activity of previously emerged adult females, and emergence of new females.

A distinct empirical relationship between precipitation and the response variable would support the hypothesis being tested (H1 or H2), even if that relationship is complicated or multimodal. On the other hand, we would interpret no significant correlation and explanatory power of predictor variables with female counts as a lack of control on mosquito abundance resulting from precipitation (the alternative hypotheses in each case). We would then conclude that anthropogenic sources of water are releasing the mosquito species from the constraints of water available from precipitation in urban contexts.

**Figure 2:**
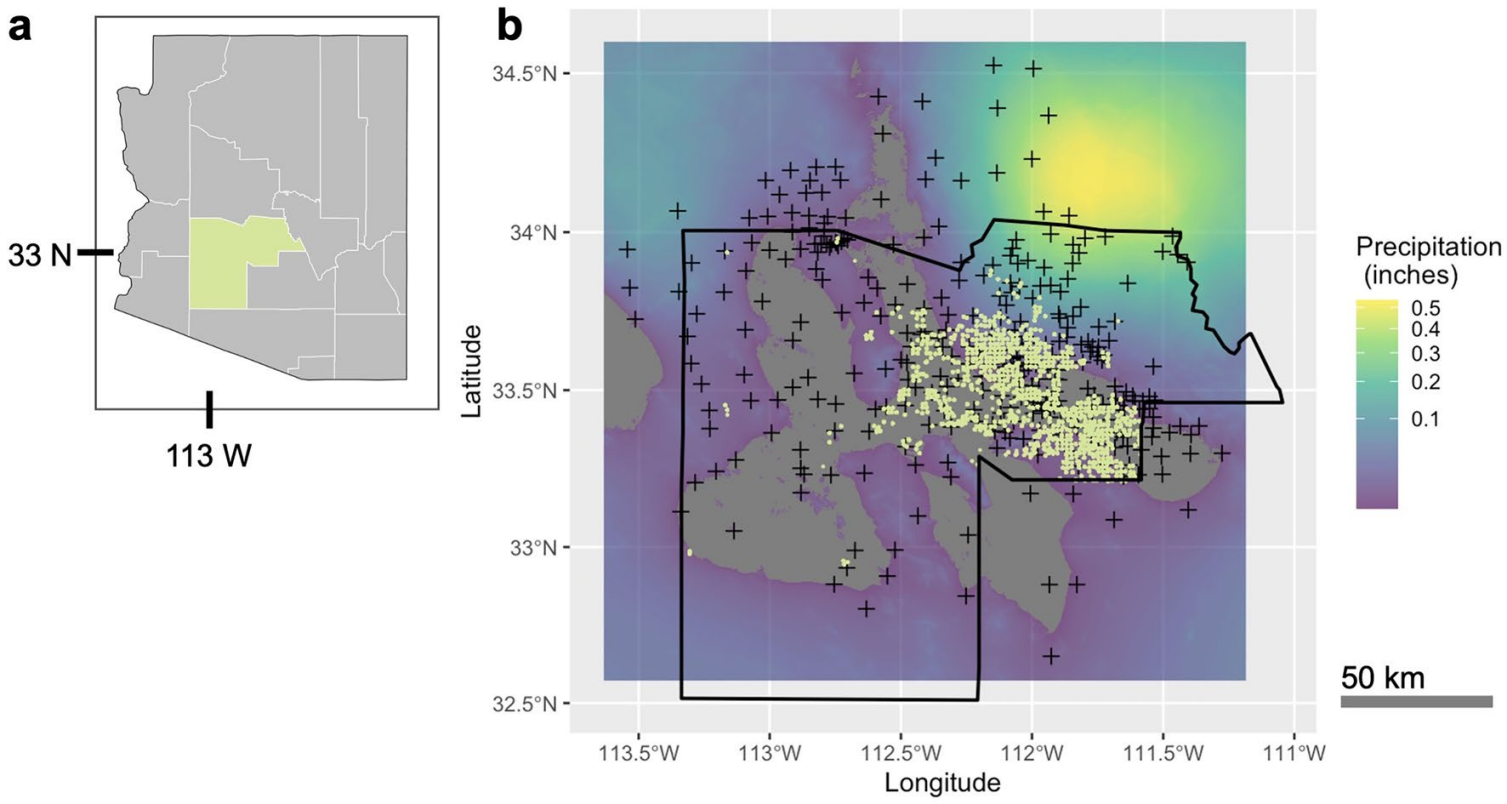
Spatially interpolated precipitation is matched to trapping station locations. Example of daily precipitation (rainfall) data for a single day, with spatial interpolation between 355 weather stations at the resolution of 10 arc-seconds (~ 300 m). The 842 traps were matched with spatially explicit, daily precipitation data from multiple rasterized layers of interpolated precipitation. This allowed us to examine how the amount of precipitation received prior to trapping events interacted to affect *Ae. aegypti* developmental period (*i.e.* the time from egg laying to emergence of the adult mosquito) and therefore the abundance of trapped adult mosquitoes. (**a**) A map of Arizona, USA is shown with Maricopa County highlighted. (**b**) In the map of Maricopa County, locations of weather stations are shown as black crosses, and trap locations are in light green. Maps were made in the *R* programming language (https://www.r-project.org/) v4.3.1^89^, with package ‘ggmap’ (https://CRAN.R-project.org/package=ggmap) v.3.0.2^93^.

We investigated these hypotheses, as well as other relationships of spatial and temporal patterns of abundance, by matching daily precipitation at the trapping locations to numbers of female *Ae. aegypti* captured each week by the Maricopa County Environmental Services Vector Control Division (Fig. 2). These data were collected from 2014 to 2016, from a network of over 660 weekly-sampled CO_2_-baited traps distributed throughout Maricopa County, with more locations added in each year (Table 1). An application of a kriging algorithm^59^ to weather station data allowed us to interpolate local conditions between measured points, generate daily precipitation data layers, and match the scale of this predictor variable to the life cycle of mosquitoes. We then reconstructed the relationship between daily precipitation amounts and timing to the eventual outcomes of each trapping event. With increased spatial and temporal resolution available from kriging, we were able to generate new insights into how mosquitoes are directly affected by precipitation at the sites where they develop, emerge, and breed.

**Table 1:** Summary of trapping effort.

Year/data	Trap type	Traps	Number of surveys	Number of traps with non-zero abundance (percentage)	*Aedes aegypti* mosquitoes trapped	Number of females (percentage)
2014	CO_2_-baited	666	28,131	3951 (14.1%)	39,915	27,208 (68.2%)
2015	CO_2_-baited	785	34,447	6051 (17.6%)	37,746	24,155 (64.0%)
2016	CO_2_-baited	794	37,901	6560 (17.3%)	44,219	32,132 (72.7%)
	BG Sentinel	19	278	105 (37.8%)	377	254 (67.4%)
Totals	*Unique traps:*	842	100,757	16,667 (16.5%)	122,257	83,749 (68.5%)

Year, number and type of trap, number of *Ae. aegypti* mosquitoes trapped, and number of and percentage of *Ae. aegypti* females from Maricopa County, Arizona, USA for 2014–2016. The number of traps with non-zero abundance are those that contained at least 1 male or female *Ae. aegypti* mosquito; those that only contained positive female counts are 15,882 traps, or 15.8% of total trapping events.

## Results

### Mosquito trapping

Maricopa County Environmental Services Vector Control Division collected mosquitoes from CO_2_-baited traps weekly and BG Sentinel traps at a total of 842 unique locations from January 2014–December 2016. Median distances between traps were determined to be 0.825 mi (1328 m) (using R package ‘raster’^60^). There were a total of 100,757 discrete trapping events, and 122,257 *Ae. aegypti* mosquitoes were collected. The majority of trapping events did not contain *Ae. aegypti*, but there were 15,882 unique trapping events with 1 or more females. Of the trapped *Ae. aegypti* mosquitoes, 68.5% or 83,749 were female (Table 1).

### Seasonal effects on the number of female *Ae. aegypti*

**Figure 3:**
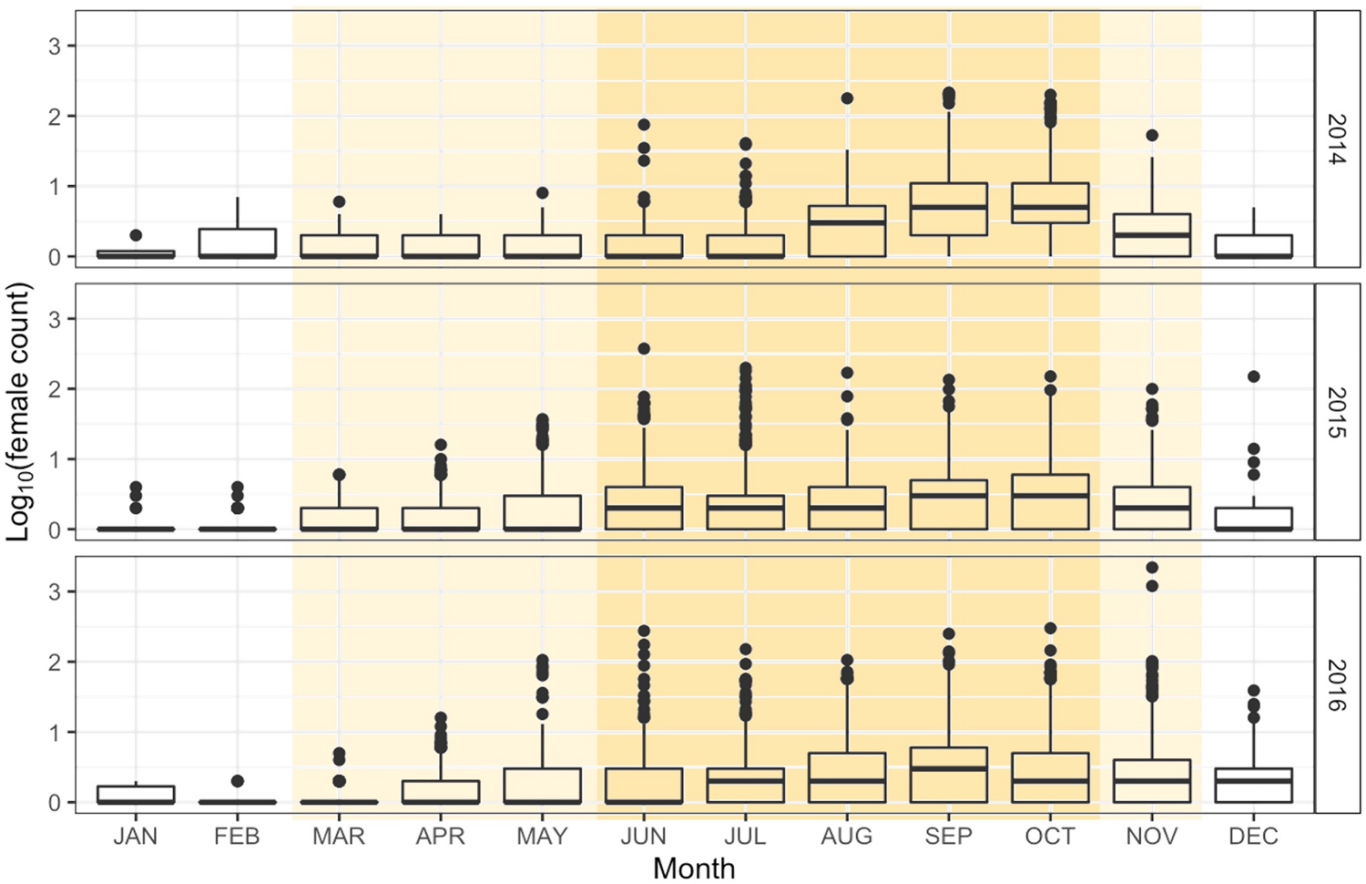
Mosquito abundance varies with season outside of laboratory-derived temperature limits. The numbers of trapped *Ae. aegypti* females are shown on a log_10_ scale by month for 2014–2016 (non-zero counts only). The yellow highlighted region represents the active season (March-November), and the “high activity” season (June-November) is shown in orange. These include all of the monsoon months (July–September). Historically, average minimum monthly temperatures are below the larval survival threshold for *Ae. aegypti* (12 °C/54°F) in December-April^65^, and exceed the laboratory-derived physiological limit for maximum temperature (35 °C/95°F) in May-September^34^, however, monthly temperature normals have been increasing in recent years. The activity period, determined empirically by Maricopa County Environmental Services Vector Control Division, was longer in duration for this time period compared to historical norms.

Female counts, a proxy measure for total *Ae. aegypti* activity and abundance and more direct indicator of disease transmission risk than total *Ae. aegypti* adults, varied seasonally. The monsoon season July–September coincides with high temperatures, reducing the generation time for the *Ae. aegypti* mosquitoes and providing additional water sources for breeding and survival. Based on historical data, we expected higher trapped abundance counts in June-October, but found high female counts both within the monsoon season, and in the surrounding months (Fig. 3) including March-November.

### Spatial autocorrelation

**Figure 4:**
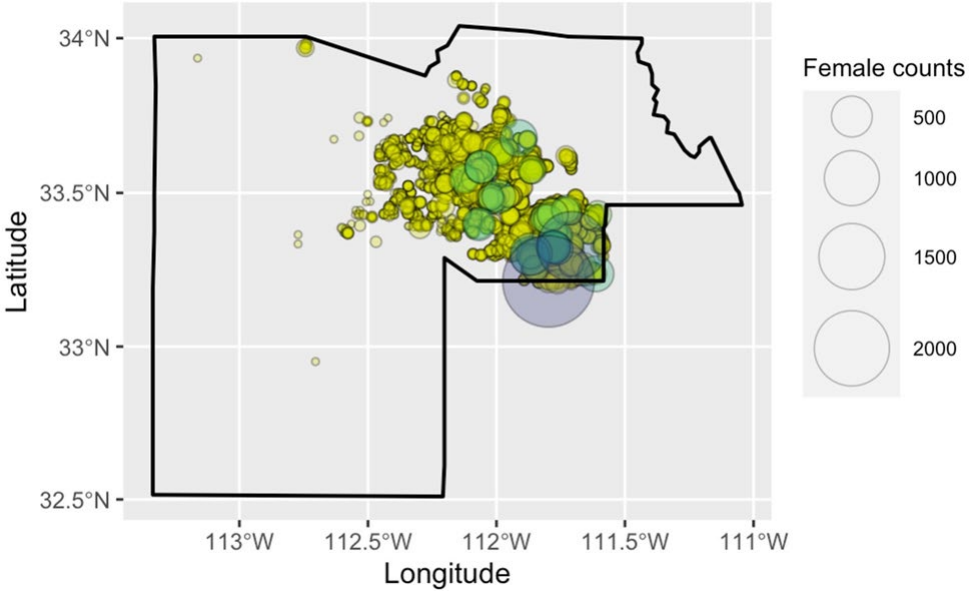
The spatial distribution of trapped mosquitoes shows extreme local differences, even within the same climate. Spatial distribution of female *Ae. aegypti* counts recorded from 100,757 discrete trapping events at 842 locations from January 2014-December 2016 in Maricopa County, Arizona, USA. The observed Global Moran’s* I*_*obs*_ = 0.055 (*sd* = 0.003; *p* < 10^–10^; expected value of *I*_*exp*_ = − 0.001) for aggregated trapped female *Ae. aegypti* over three years (765 traps surveyed 60 times each) indicates that mosquitoes are highly clustered in space compared to a uniform distribution. A color ramp is used to better visually distinguish between point sizes. Maps were made in the *R* programming language (https://www.r-project.org/) v4.3.1^89^, with package ‘ggmap’ (https://CRAN.R-project.org/package=ggmap) v.3.0.2^93^.

Our spatial analyses focused on female counts at 765 equally sampled trapping locations, with counts aggregated over 3 years. We calculated the Global Moran’s* I* statistic to understand the clustering of events over the study area extent (Fig. 4). We calculated *I*_*obs*_ = 0.055 (*sd* = 0.003; *p* < 10^−10^), with an expected value of *I*_*exp*_ = -0.001, indicating that *Ae. aegypti* trap locations and/or trap counts are significantly highly clustered in space compared to a baseline of a random distribution. As trapping locations are placed mostly on a regular grid, this implies high spatial autocorrelation of trap counts.

### Determining the relationship of daily precipitation to trap outcomes

Our precipitation analyses were aimed at gaining insight into which variables best predict *Ae. aegypti* presence and abundance. For comparison, we calculated average precipitation across all observations at the trap locations, and found that it is low and nearly constant (0.025 in., var ~10^−5^ in.). Because this single, regional average of precipitation cannot explain the highly variable patterns of local mosquito abundance, we investigated the localized, daily precipitation patterns, including daily and cumulative precipitation.

To determine whether or not daily precipitation variables were likely to affect trap outcomes, we performed preliminary analyses. We graphed daily precipitation from kriging and resulting counts of mosquitoes, with zeroes separated from non-zero counts (Supplementary Material: Fig. S1), with no clear pattern apparent. We then calculated correlation coefficients for daily precipitation and female presence, and separately, female abundance (Supplementary Material: Fig. S2). These analyses showed that prior days’ precipitation had differing effects, both in magnitude and direction, on trap outcomes. These preliminary analyses are similar to a Cross Correlation Function, in that each approach is used to compare two time series and identify which lagged variables from one time series can be used to predict values from the other time series. In CCF analysis, the larger environment drives the patterns in the time series on the same calendar dates, but here it was necessary to realign the time series to the emergence date. The correlation coefficients form an intermediate step to establishing that the days have different effects on outcomes, to justify the more rigorous LASSO^61^ (least absolute shrinkage and selection operator) modeling.

### Modeling prior precipitation, female presence, and counts

For hypothesis H1, we tested whether or not adult mosquito presence and abundance is limited by *daily precipitation from days prior to and after the egg-laying period*. With *a priori* knowledge of the life cycle of *Ae. aegypti* at higher environmental temperatures, we expected clear increases in counts on days 7–15 days following precipitation events, and decreases 5–20 days following precipitation events. Based on LASSO modeling described below, we did find that the daily patterns of precipitation mattered to presence and abundance, but we did not find the expected pattern. For hypothesis H2, which tests whether or not adult mosquito presence and abundance (both emergence and activity of previously emerged adult females) is limited by *cumulative precipitation both before and after the egg-laying period* in the vicinity of the trapping location, we calculated accumulated precipitation at each trapping location (counts including zeros are shown in Supplementary Materials: Fig. S3). We investigated the effects of precipitation for two cumulative precipitation thresholds, 10 and 20 days leading up to trap collection, for each trapping event. We compared the explanatory power of daily precipitation information with that of cumulative precipitation for the two cumulative precipitation thresholds through multi-model comparisons.

For mosquito presence in traps, LASSO logistic regressions show we obtain better results when using all the daily precipitation predictors instead of combining them into cumulative predictors, at a small cost in model complexity (Table 2). However, the gains are not large, and the AUC-ROC value of the best model is <0.75, indicating a poor model fit overall. Several daily precipitation variables are consistent with zero (days 1, 5, 9, 13, and 20) and may have no effect on ultimate trapping success. Precipitation 2 days before trap collection had a strong, negative effect on female presence in traps, and day 8 has a weaker effect. All other days had a positive correlation with presence of female mosquitoes.

**Table 2:** Multiple model comparisons for presence and abundance of female *Ae. aegypti*, with daily and cumulative precipitation predictors.

Model	Metric	Estimate
Logistic Presence intercept-only (null model)	AUC-ROC	0.500
Logistic Presence (days + 10 days cumulative precipitation)	AUC-ROC	0.543
Logistic Presence (days + 20 days cumulative precipitation)	AUC-ROC	0.582
**Logistic Presence (Daily precipitation)**	**AUC-ROC**	**0.656**
Poisson abundance intercept-only (null model)	RMSE	4.850
Poisson abundance (days + 10 days cumulative precipitation)	RMSE	4.850
**Poisson abundance (days + 20 days cumulative precipitation)**	**RMSE**	**4.832**
Poisson abundance (Daily precipitation)	RMSE	4.844

Models for both presence and abundance are compared using each day’s daily precipitation as predictors, as well as cumulative precipitation with a 10 or 20 day horizon. Best-supported models are chosen through LASSO (least absolute shrinkage and selection operator) regressions and shown **in bold**. Logistic models are performed for presence and evaluated with area under the curve of the receiver-operating characteristic curve (AUC-ROC; with larger values corresponding to better model fits), while abundance models are fit to Poisson distributions and evaluated with root mean squared error (RMSE; with lower values corresponding to better model fits). Precipitation variables increase model performance for presence models, but abundance models all perform similarly, indicating poor model performance with only these variables.

We constructed similar models for abundance with Poisson distributions. LASSO regressions in this case show that daily precipitation combined with cumulative precipitation over 20 days is the best model for mosquito abundance, however, the gains over the other models including the null, intercept-only model are minimal, generally indicating poor model fit (Table 2). This implies that the LASSO modeling may not be appropriate for this application and other types of models should be investigated, or that major, driving variables, such as temperature, must be included for meaningful models. With only kriged precipitation data, we cannot resolve this issue, and similarly kriged and locally interpolated temperature data would be required for this kind of investigation.

We find that for H1, daily precipitation variables affect presence outcomes and may influence abundance outcomes. We find that for H2, cumulative precipitation does not affect presence outcomes, but 20-day cumulative precipitation may affect abundance. Models for both presence and abundance containing either 10-day or 20-day cumulative precipitation by themselves did not perform as well as models containing daily precipitation.

**Figure 5:**
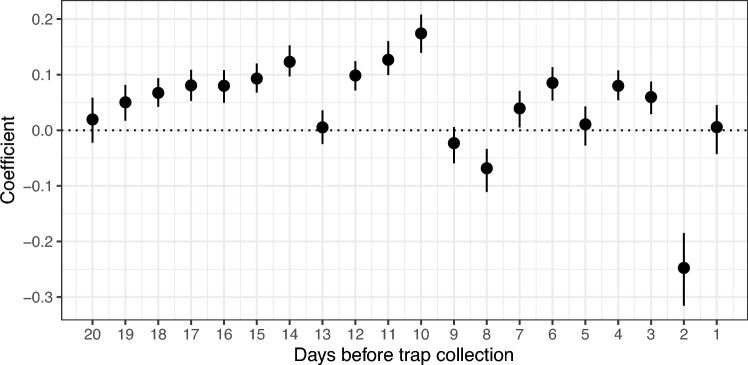
Modeled coefficients of prior precipitation effects on female *Ae. aegypti* presence (intercept excluded). A best-performing LASSO model for female mosquito presence contains all daily precipitation variables and no cumulative precipitation variables. Although the estimate for the intercept is not shown, its coefficient is − 1.86 (95% CI − 1.89, − 1.82), which is by comparison a larger effect than any single daily precipitation variable. Estimated coefficients are shown with 95% bootstrap confidence intervals, constructed from 1000 replicates. Several daily precipitation variables are consistent with zero (days 1, 5, 9, 13, and 20) and may have no effect on ultimate trapping success. Precipitation 2 days before trap collection has a strong, negative effect on female presence in traps, and day 8 has a weaker effect. All other days have a positive contribution to mosquito presence, with the largest effect 10 days before trap collection.

Overlaying the correlations with the life cycle as presented in Fig. 1, we see that precipitation events occurring during the early larval stages or at the time of adult emergence can prevent the presence of mosquitoes in traps entirely, and at other times during mosquito development, precipitation has no effect, or a positive effect on mosquito presence (Fig. 5).

### Estimating the minimum influence of anthropogenic water on mosquito abundance

To assess the potential importance of anthropogenic water sources on driving patterns of mosquito abundance and activity, we examined all trapping events that produced at least one positive female count (“positive traps”; 15,882 observations). We then examined which of these trapping events were associated with little to no measurable precipitation in the 20 days prior to collection. Precipitation-free periods would not meet the inundation requirements for developing eggs in containers, and therefore, mosquitoes that are trapped in these periods would require anthropogenic water sources for their development.

We determined a “low precipitation threshold” by examining trap outcomes for which additional precipitation did not affect the maximum count in the trap. The minimum precipitation threshold was determined to be 0.02 inches (27.6% of all trapping events; max female count = 175), below which all counts were approximately or actually equal. These traps contain 10% of total trapped females. Above 0.02 inches of precipitation, mosquito counts increased rapidly. We therefore estimate a background population of at minimum 10% of *Ae. aegypti* that are not dependent upon precipitation.

## Discussion

Understanding conditions that lead to greater presence and abundances of *Ae. aegypti* is critical to controlling the spread of arboviruses to humans. We examined how the amount of preceding precipitation interacts with the *Ae. aegypti* life cycle to affect the abundance of trapped female mosquitoes. We found that the active season of *Ae. aegypti* in urban Maricopa County, Arizona as determined by the Maricopa County Environmental Services Vector Control Division was longer in duration during this time period compared to historical norms (Fig. 3). Summer temperatures exceed laboratory survival tolerances for *Ae. aegypti*, and their increased abundance during these periods may point to dispersal to suitable microhabitats^62^. We also examined the clustering of female counts at 765 equally-sampled locations over three years using global Moran’s *I*, and found high clustering compared to a random baseline. Areas where exceedingly high counts (>500 individuals in a single trap) appear on the map indicate where it could be productive to search for larval development sites and target control efforts. Here, the specific locations of large outbreaks might be associated with clogged and unmaintained storm drains (K. Walker, *unpublished data*) serving as breeding habitat, as have been found to support *Ae. aegypti* populations in other regions^50,63^.

The interaction between precipitation and human-built structures may indeed be driving the emergence of a major disease vector in this region^64^, but more investigation is warranted, given that human density, differences in land usage, and site-specific, targeted mosquito control efforts may also influence these counts (see https://maricopa.maps.arcgis.com/apps/webappviewer/index.html?id=c00b3ecbb3344ca2930a30b978184ddd; accessed November 2023). Tracking the large amounts of outdoor water generated by human uses (70% of all human uses estimated; see https://www.azwater.gov/conservation/public-resources) could lead to validation of some results presented here, as well as better mosquito control efforts.

We expected that cumulative rainfall over the period of 20 days prior to trap collection should influence the presence and abundance of trapped mosquitoes, and also tested whether or not daily precipitation leading up to trapping had any influence on trap outcomes. Through correlation analyses, we found differing influences, in terms of both magnitude and sign, of each prior day’s precipitation on subsequent female mosquito presence and counts (Supplementary Material: Figure 2).

Although we expected to find that both low and high precipitation limits *Ae. aegypti* numbers, we instead found a relatively flat relationship of abundance with cumulative precipitation over 20 days (Supplementary Material: Fig. S3). LASSO models revealed a potential role for cumulative precipitation to be driving patterns of abundance, but not mosquito presence. Our preliminary correlation coefficient analyses showed relatively clear patterns of differing daily effects of precipitation on both presence and abundance (Supplementary Material: Fig. S2), only a subset of these patterns were supported with the applications of LASSO regressions. LASSO models demonstrated that daily (but not cumulative) precipitation influences mosquito presence, with suppression of mosquito presence when precipitation occurs on days 2 or 8 before trap collection. These results imply interruption of critical life cycle events, such as larval development or selection of oviposition sites of the parent generation (day 8 prior to trap collection), or emergence (day 2 prior to trap collection). Precipitation on many other days, most notably day 10 before trap collection, positively influence presence. Cumulative precipitation on a 20-day time horizon (in addition to daily precipitation variables) may be important for predicting mosquito abundance, but not presence. However, we also find that LASSO models for female *Ae. aegypti* presence and abundance are insufficient when only containing precipitation variables. LASSO models may perform better with the inclusion of local temperature, humidity, and other important environmental variables. We conclude that neither daily nor cumulative precipitation by themselves explain mosquito presence or abundance, and better models may need to include predictors known to influence mosquito populations.

We note that on occasions when rain events happen concurrently with the operation of the trap, there may be multiple reasons for reduced counts. Trap effectiveness can be disrupted if the attractant CO_2_ column is dissipated by rain or accompanying wind, making it difficult for the mosquito to find the location of the trap (thereby reducing catches), or local temperature fluctuations during rainfall events lower the sublimation rate of the dry ice that provides the CO_2_ bait. Fewer counts are recorded when there are wet mosquitoes in the trap, because these are often damaged and not easily identified. However, it may be generally true that precipitation interferes with successful emergence of adults, which could explain why precipitation on day 8 before trap collection (corresponding to one generation) correlates negatively with mosquito presence.

We did not find direct evidence of larval flushing, and future efforts may need to look at large precipitation events in isolation to determine if this is a special factor in determining mosquito abundance. Other future research may focus on another precipitation pattern known to affect mosquito abundance, dry-rainy frequency^13,14^, which might be testable with this method if the study period was lengthened beyond 20 days of precipitation.

Where patterns of *Ae. aegypti* emergence and capture are not affected by low precipitation (as is true for ~28% of traps with any female *Ae. aegypti* in them, and ~10% of the total number of trapped females in this study), we conclude that anthropogenic water sources provide the likely breeding habitat for *Ae. aegypti* populations^41,46,49,62^. However, the true dependency of the population on anthropogenic water for all reasons (e.g., microclimate modification, survival, breeding) is likely much higher than 10%.

Along with precipitation, it is well established that both temperature and relative humidity affect larval development rate, adult survival and activity, with increasing temperatures leading to faster development times^40,65^. Relative humidity might show similar trends due to increased movement and survival among eggs and adults^39,66^, but this relationship may be complicated by the interaction with mosquito fecundity^67^.

We looked uniquely at precipitation and trap data that were pooled across all trapping dates and locations, and re-indexed these data to a 20-day data set. Although this is a powerful way of determining positive or negative influence of precipitation events during the development of the mosquitoes, we note that fixed factors like land use and human density would broaden the variation around the estimates of the influence of daily precipitation, while factors that vary in time, such as temperature and humidity, would blur the effect of which particular days were significant. We again conclude that attempts to model presence and abundance of mosquitoes given microclimate-scale data will benefit from the incorporation of additional variables measured or interpolated at the same scale, such as temperature^68^.

The application of kriging to the problem of predicting mosquito abundance allows us to gauge the relative effects of precipitation and anthropogenic water sources in this region. Taking this further, urban planners might incorporate this information for smarter use of urban water that does not directly lead to increased mosquito abundance. If kriging methods were also applied to local-scale variations in temperature and humidity, along with their interaction with precipitation in the environment, we could gain further insight into what makes up the microclimates that drive establishment patterns and abundance of *Aedes* mosquitoes across very different climates^21^. In this way, the search for commonalities among regions from a “microclimate perspective” might resolve some of the issues surrounding how to accurately predict *Aedes* invasions into new regions including humid, tropical regions, and the urbanized Sonoran Desert. These results might then be applied where fine-grained environmental data is collected by satellites, as data are abundant and not yet used to their full potential in disease ecology^26^.

This study demonstrates that kriging with weather station precipitation data can resolve certain questions about precipitation and the *Ae. aegypti* life cycle in the presence of anthropogenic water sources, and outside of a laboratory setting. Kriging with multiple variables could bring “big data” science (and the computational storage and power required) to bear on questions of disease vector expansion, which has extreme significance to global human health. Broader applications of kriging with multiple variables could serve as a useful decision support tool for disease vector control in urban settings, as microclimates can be modified more easily than climate normals.

Beyond understanding the dynamics of disease vector populations and disease ecology applications, the application of kriging in conjunction with organismal life cycles will be useful in the context of conservation planning^69^. The ability to examine highly localized and variable conditions that precede ecological events and the distributions of seasonal phenological phenomena^70^ will have broad applicability across ecological questions and ecosystems. Kriging may become a valuable planning tool for many organismal-environment problems, including predicting outbreaks of tree-killing beetles^71^, understanding drivers of arthropod declines^72^, anticipating changes to water bodies after precipitation^73,74^; and making more granular predictions of effects of global change on organisms such as amphibians^75,76^, those dependent on specialized interaction partners^77,78^, and rare and biogeographically limited species^79^.

## Methods

### Study region

The current range of *Ae. aegypti* now includes almost all populated areas in Maricopa County, overlapping with high and increasing human population density, and land use modifications arising from increasing urbanization and suburbanization. Maricopa County spans 9,224 square miles (~23,890 sq. km), and contains 27 cities and towns including the metropolitan Phoenix area, and all or part of five tribal nations’ federally designated reservation lands. Maricopa County includes 4.552 million people as residents as of the 2022 census.^80^

Maricopa County is located in the biogeographic region of the Sonoran Desert, which has five seasons (winter, spring, fore-summer occurring in May and June, summer monsoon in July and August, and fall), and experiences two seasons of rainfall (winter and summer monsoon). Climate normals for the area from 1981 to 2010 include a mean minimum temperature of the coldest month (December) of 44.8 °F (7.1 C); mean maximum temperature of the warmest month (July) of 106.1 °F (41.2 C); and average annual precipitation of 8.03 inches (20.4 cm), with 25% of the rainfall occurring in July and August, and approximately half of the precipitation arriving throughout December-March (retrieved from the NOAA National Center for Environmental Information via the National Weather Service Forecast Office website: https://w2.weather.gov/climate). Recent climate change (post-2010) is increasing the maximum temperatures of the warmest months, as well as the average temperature, and the number of days over 100°F. High and low temperatures in this region are known to exceed laboratory-derived physiological limits for *Ae. aegypti*^27,34,65^.

### Mosquito trapping protocols

Mosquitoes were trapped by Maricopa Environmental Services–Vector Control Division at trapping sites established throughout the urban and suburban areas of Maricopa County. Traps were placed at a density of one trap per square mile ( ~ 1610 meters), while accommodating urban structures (Fig. 2). Additional traps were placed temporarily in response to complaints about mosquito densities and in areas with reported human arbovirus cases. Standard CO_2_-baited traps (Silver 2007) were established at 842 unique sites (Table 1). Trapping occurred once per week at each site throughout the year. Traps were hung ~1 m from the ground and left overnight to collect mosquitoes, which were then identified to species in the Maricopa County Environmental Services Vector Control Division laboratory, sorted by sex, and counted.

The Maricopa County mosquito surveillance program was designed primarily to monitor *Culex* mosquitoes associated with West Nile Virus transmission. The current recommended methods for collecting adult female *Ae. aegypti* are the BG-Sentinel trap, the Autocidal Gravid trap, or backpack aspiration^81,82^, however, the CO_2_-baited traps used to trap *Culex spp.* do attract *Ae. aegypti* mosquitoes, and trap counts are expected to reflect the true variability across the geographic area. Of the total trapping effort, BG traps were only used in 19 locations in 2016, for a total of 278 discrete trapping events (and 254 total *Ae. aegypti* females). These were excluded from analyses.

### Environmental data

Daily precipitation data were downloaded from the Flood Control District of Maricopa County (FCDMC) weather stations (*n*=355) (at https://www.maricopa.gov/625/Rainfall-Data). While this is a high density of weather stations compared to other similar sized regions in the US, the weather stations are clustered together, and geographic coverage was not complete. Values for daily precipitation for areas not directly measured were therefore spatially interpolated between weather stations using kriging. Kriging is a standard algorithm used to predict values in regions where data has been collected from the surrounding regions^59^, that is used to search out parameters associated with known covariance data, and apply this information to new or unknown regions. In this study, a kriging algorithm was trained on daily precipitation data using elevation as a covariate using R ‘automap’ package^83^. We adopted an automatic kriging function where a variogram model was fit using predefined models (spherical, exponential, Gaussian, Matern) with the default settings. The initial sill was estimated as the mean of the maximum and median of the semi-variance. The initial range was defined as 0.1 times the diagonal of the bounding box of the data, and the initial nugget was defined as the minimum semivariance. Elevation data were downloaded from the USGS National Elevation Dataset (http://ned.usgs.gov) and resampled to the resolution of 309 by 371 meters.

Kriging was carried out on daily precipitation data for all days from October 2012-September 2017, to create spatially resolved, daily rasters for the study region (Fig. 2)^84^. A relevant subset of these rasters were then matched to each individual trapping event, from 1 to 20 days prior to the event, inclusive. Interpolated precipitation data was then extracted by location. Dispersal distances of *Ae. aegypti* are known to be much lower than the distances between traps in the network. Therefore, information derived from kriging should better represent the conditions relevant to life cycle events of the mosquitoes than regional averages for precipitation.

The methods presented in this manuscript track all mosquito trapping events over space, realign the precipitation time series by presumptive mosquito emergence date, and correlate the emergences to precipitation. This method is appropriate in light of the spatial heterogeneity of landscape factors such as differences in human density, agricultural uses, and targeted mosquito control (which is performed occasionally for *Ae. aegypti* after large trapping events have occurred, and not over the entire trapping region), as those will not affect the estimation of the precipitation’s effect on presence or abundance of mosquitoes, but rather, will only increase the variance on modeled fits.

### Spatial autocorrelation

We limited statistical analyses to female *Ae. aegypti* mosquitoes, as only females transmit arboviruses. The number of individual females in each trap (“counts”) is used as the proxy metric for the activity of female *Ae. aegypti*. We first investigated the spatial autocorrelation of mosquito counts across the study area on aggregated female counts trapping locations over all three years. Global Moran’s *I* measures spatial autocorrelation of events based on their location and values, and reports whether they are clustered, dispersed, or random in space. To calculate Global Moran’s *I*, we required a set of points that were sampled equally, so we limited the analysis to 765 locations with CO_2_ traps that were sampled 60 or more times (those with >60 trapping events were limited to a random draw of 60 events).

### Seasonal effects on the number of female *Ae. aegypti*

We examined trap counts over months, and expected higher counts in trapping events that occur during June-October, as average minimum monthly temperatures have historically fallen below 15 °C outside this range. Temperatures of 12–15 °C and below are known to negatively affect adult female mosquito survival, and severely limit successful oviposition^65,85,86^. However, microclimates in Maricopa County^62^ can mitigate non-ideal temperature and precipitation conditions for mosquito development and lead to low emergence numbers, and temperature normals are changing rapidly in this region. We therefore included trapping events from all months in our analyses.

### Determining the relationship of daily precipitation to trap outcomes

To understand the strength and direction of the effects each day’s precipitation ultimately had on trap outcomes, we first examined the daily precipitation relationship to female presence. Because repeated sampling from a single trap within a 20 day period would cause repeated values of precipitation to show up at 7-day intervals (which is coincidentally the approximate development period for an *Ae. aegypti* mosquito), we included a step of thinning the data. Only one trapping event from each location per month was used for all presence and abundance analyses. Data points were ordered by calendar date and selected from early in the month to prevent overlapping 20-day periods.

To analyze each day’s effect on subsequent mosquito presence or abundance, modeled presence or abundance as a function of a single day’s precipitation, and extracted a correlation coefficient. For mosquito presence, we fit an independent logistic model for each day and its relationship to trap outcomes. Similarly for abundance, we assessed the correlation between each day’s precipitation and its relationship to trap counts (Supplementary Material: Fig. S2). Once we could establish that the precipitation data plausibly had different effects on trap presence and female count outcomes, we were then able to justify more rigorous modeling. LASSO models were then carried out for both presence and count models, as described below.

### Modeling prior precipitation, female presence, and counts

Considering all precipitation data together, we were able to analyze multiple relationships between prior precipitation and the number of *Ae. aegypti* collected from traps, with daily precipitation for 20 days leading up to trap collection, and with cumulative precipitation for 10 days and 20 prior to trap collection). Presence of females was modeled separately from total abundance of females.

For both presence and abundance models, we used LASSO regressions to eliminate variables from four models: a null, intercept-only model; a full model containing all daily precipitation predictors; a full model with all daily precipitation predictors and cumulative precipitation for the full 20 days leading up to trap collection; and a similar full model with a 10-day horizon for cumulative precipitation leading up to trap collection instead of the 20 days. LASSO regressions minimize the total summed coefficients of the predictors, and have the ability to drop predictors entirely, resulting in lower model complexity. For the cases of both the presence and the abundance models, we divided sites into training and testing sets so that 75% of observations are assigned to the former and 25% to the latter. For the regularized LASSO regression, we normalized the predictors in the abundance model so that the variable coefficients would be comparable, but the presence model did not improve with increasing the regularization penalty tuning parameter, so the parameter was set to zero for the final model comparisons.

We modeled female presence and absence with LASSO logistic models, and compared them with AUC-ROC (area under the receiver-operator characteristic curve) statistics, implemented in ‘tidymodels’^87^ in the *R* programming language. Similarly, we modeled abundance with multiple models, fitting each relationship with a Poisson distribution. We evaluated the abundance models by evaluating their root mean squared errors (RMSE).

### The influence of anthropogenic water sources on mosquito abundance

Finally, background levels of mosquito emergence with little or no measurable precipitation in the preceding 20 days were used to quantify the importance of anthropogenic water sources in driving mosquito abundance. Here we note a technical definition for “little to no measurable precipitation.” The values that result from kriging and spatial interpolation of weather station precipitation data can take on a range of values, including those that are small but negative (and therefore do not correspond to realistic precipitation predictions), and those that have exceedingly small, positive values (example from data: “2.252000e-10” inches). As part of this analysis, all precipitation values obtained by kriging were cleaned such that negative values, as well as values that fell below the threshold of measurable precipitation were set to zero. Small, positive values under 0.01 inches of precipitation are below the threshold of measurable precipitation according to the National Weather Service, which is part of the USA’s National Oceanic and Atmospheric Administration (https://www.weather.gov/ajk/ForecastTerms; accessed February 12, 2022)^88^. The definition of “little to no precipitation” for data interpolated by kriging therefore included true zeros (reset from negative values), and also included those values that were close enough to zero to fall below the measurable precipitation threshold.

Data were cleaned and analyzed in the *R* programming language (v.4.3.1)^89^ with the packages ‘dplyr’^90^ and ‘raster’^60^, and ‘lme’^91^. Global Moran’s* I* was calculated using package ‘ape’ (v5.0)^92^. Mapping of data was carried out with the package ‘ggmap’^93^.

### Supplementary Information

Supplementary Information.

## Data Availability

Details of trap information, including locations of traps, is available online at: https://maricopa.maps.arcgis.com/apps/webappviewer/index.html?id=c00b3ecbb3344ca2930a30b978184ddd. Weather station precipitation data are available through the Maricopa County website at: https://www.maricopa.gov/625/Rainfall-Data, and climate normals for the region are available through the NOAA National Center for Environmental Information via the National Weather Service Forecast Office website: https://w2.weather.gov/climate. Rasterized daily precipitation data layers generated for this project are available at Zenodo: https://doi.org/10.5281/zenodo.5422729. The public, online repository https://github.com/iskanderun/maricopamosquitoes contains *Ae. aegypti* mosquito male and female trap counts for Maricopa County, Arizona, USA from 2014 to 2016, including type and approximate location of traps, date of trap collection, as well as the *R* programming language software script containing the function that associates the trap location with precipitation data from single, previous days, or cumulatively for multiple previous days. Resolution of the locations of traps has been coarsened to protect currently operating equipment.
