# Supplementary material for: Defining the roles of local precipitation and anthropogenic water sources in driving the abundance of Aedes aegypti, an emerging disease vector in urban, arid landscapes
Source: Sci Rep. 2024 Jan 24;14:2058. doi: 10.1038/s41598-023-50346-3 (PMC10808563; doi:10.1038/s41598-023-50346-3)
Supplement: Supplementary file 1 — Supplementary Information. [file 41598_2023_50346_MOESM1_ESM.pdf]

Supplementary Material for:

Defining the roles of local precipitation and anthropogenic water sources in driving the abundance of *Aedes aegypti*, an emerging disease vector in urban, arid landscapes

Erica A. Newman[1,2,*]†, Xiao Feng[3], Jesse D. Onland[4], Kathleen R. Walker[5], Steven Young[6],Kirk Smith[6], John Townsend[6], Dan Damian[7], Kacey Ernst [8]

*correspondence to: newmane@berkeley.edu
† present address: University of Texas at Austin, Department of Integrative Biology

[1] Department of Ecology & Evolutionary Biology, University of Arizona, Tucson AZ 85721, USA

[2] Department of Integrative Biology, University of Texas at Austin, Austin, TX, 78712, USA

[3] Department of Geography, Florida State University, Tallahassee, FL 32306, USA

[4] Independent analyst, Kitchener, ON, Canada

[5] Department of Entomology, University of Arizona, 1140 E South Campus Drive, Forbes 410, Tucson, AZ 85721, USA

[6] Maricopa County Environmental Services Vector Control Division, 3220 W Gibson Ln, Phoenix, AZ 85009

[7] Maricopa County Office of Enterprise Technology, 301 S 4th Ave #200, Phoenix, AZ 85003, USA

[8] Mel and Enid Zuckerman College of Public Health, University of Arizona, Tucson, AZ 85721, USA

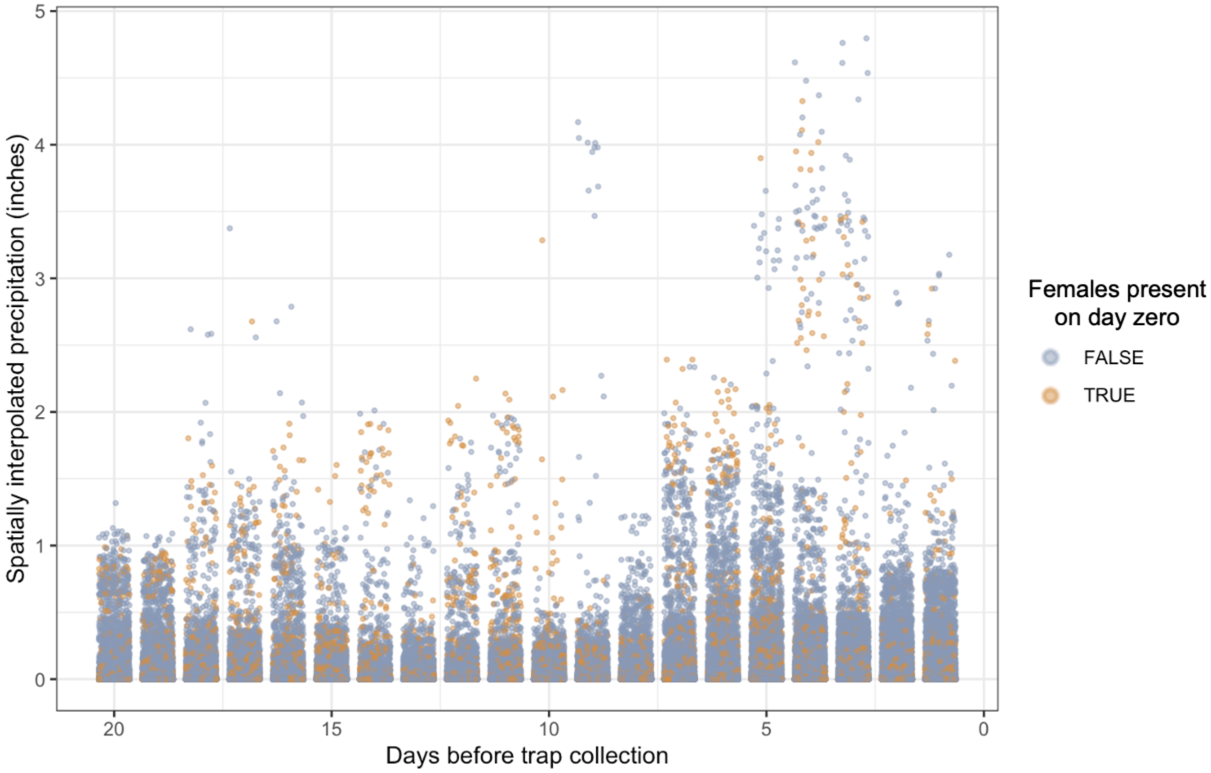

Figure S1. Counts of female *Ae. aegypti* (including zeroes) in traps, and the associated daily precipitation prior to trap collection. This graph shows the outcomes of 26,032 trapping events, for a subset of the trapping events that occur at each location no more than once per month. Both positive counts (orange) and zero counts (blue) are shown. Heavy rains on day 9 prior to trap collection lead to zero trapped females, possibly indicating interference with a developmental stage that leads to zero adult female mosquitoes in traps 9 days later.

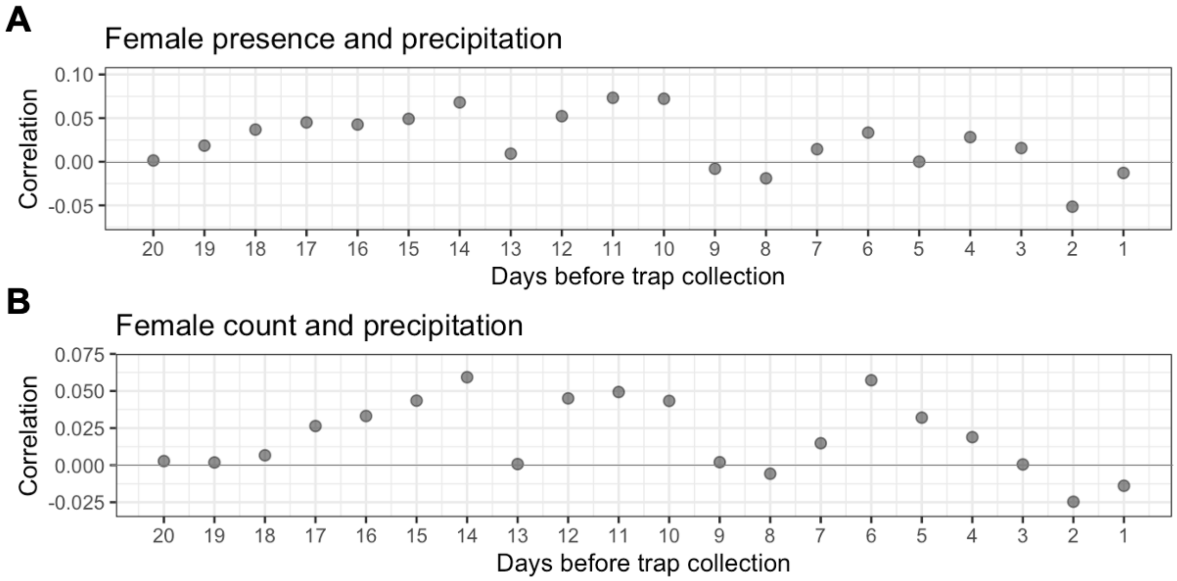

Figure S2. Correlations between female mosquitoes and prior precipitation. Presence/precipitation correlations were determined by simple logistic regressions performed for each day, after realignment of the time series by emergence date. Female count/precipitation correlations are simple bivariate correlations by day. This preliminary analysis is similar to a cross-correlation function, and was carried out to identify possible lags between precipitation and eventual mosquito trap data. (**A**) Precipitation on days 1, 2, 8, and 9 before trap collection is negatively correlated with the presence of female mosquitoes (as measured by the number of positive traps). Precipitation on days 5 and 20 before trap collection has no effect on female presence, and all other days' precipitation are positively correlated with mosquito presence. Panel (**B**) shows a similar pattern for precipitation and female counts in traps. Here, precipitation on days 1, 2, and 8 prior to trap collection is negatively correlated with female counts (interpreted as precipitation interfering with development and suppressing abundance), and days 3, 9, 13, 19, and 20 prior to trap collection show no correlation between precipitation and female counts. Precipitation on all other days increases mosquito abundance. Note that the *y*-axis represents a different scale in each panel, and the *y*=0 line is darkened in each case as a visual aid.

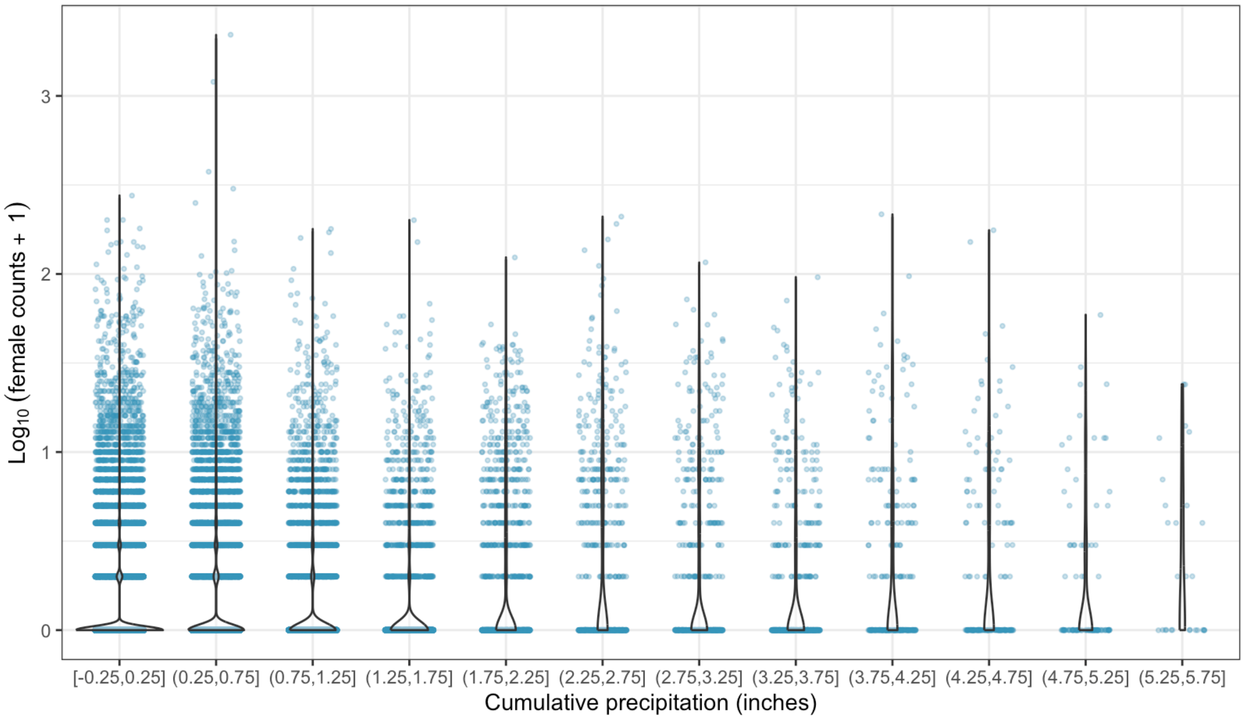

Figure S3. Female *Ae. aegypti* counts (including zeroes) are shown against binned, cumulative precipitation. Female counts in traps, transformed as $\log_{10}$(female counts + 1), associated with binned, cumulative precipitation in the 20 days prior to trap collection. There are n=100,757 unique trapping events shown in the figure. Precipitation data is from spatially interpolated, kriged raster layers, and may overestimate actual precipitation values. Violin plots overlay data points representing $\log_{10}$(female counts + 1) associated with different total amounts of precipitation over 20 days shows the predominance of trapping events that contained zero females. Of the trapping events, 15,882 or 15.8% of total trap events contained females, and 84,875, or 84.2% did not.
